# Local Delivery of Irinotecan to Recurrent GBM Patients at Reoperation Offers a Safe Route of Administration

**DOI:** 10.3390/cancers16173008

**Published:** 2024-08-29

**Authors:** Christopher McConville, Sarah Lastakchi, Ali Al Amri, Desire Ngoga, Oluwafikayo Fayeye, Garth Cruickshank

**Affiliations:** 1 School of Pharmacy, Institute of Clinical Sciences, College of Medical and Dental Sciences, University of Birmingham, Birmingham B15 2TT, UK; 2 Pediatric Neurosurgery, The Bristol Royal Hospital for Children, Bristol BS2 8BJ, UK; 3 Department of Neurosurgery, University Hospitals Birmingham, NHS Foundation Trust, Birmingham B15 2GW, UKgscruickshank@btconnect.com (G.C.)

**Keywords:** recurrent glioblastoma, irinotecan, intraparenchymal administration, local delivery

## Abstract

**Simple Summary:**

The survival of glioblastoma (GBM) patients remains at just 12–15 months with a 5% 5 year survival despite them undergoing a harsh and brutal treatment regimen involving surgery, radiation and chemotherapy. This is because GBMs are impossible to completely resect and almost always recur at the borders of the resection margin, while the presence of the blood–brain barrier limits the amount of chemotherapy that can access the brain, requiring the patient to receive high and extremely toxic doses of chemotherapy. In this study, we demonstrate that local delivery of the chemotherapeutic drug irinotecan directly into the border of the resection margin offers a safe route of administration with none of the normal side effects associated with traditional chemotherapy.

**Abstract:**

Glioblastomas are impossible to completely resect and almost always recur at the borders of the resection margin. There is no established chemotherapy regimen available to patients who recur, while systemic treatment is hampered by the blood–brain barrier. Here, we report on the first evaluation in humans of the intraparenchymal injection of irinotecan into the resection cavity after surgical resection of recurrent glioblastoma patients. The cytotoxicity of irinotecan was compared to SN-38 in primary cells from recurrent glioblastoma patients. Irinotecan was injected at multiple (~30) sites of the resection cavity wall at a depth of 3 to 5 mm. SN-38 was more cytotoxic than irinotecan at concentrations below 1 µM due to enzyme kinetics. The intraparenchymal administration of irinotecan was safe, with good wound healing and an absence of swelling, inflammation, or pseudo-abscess formation. The median survival post irinotecan administration was 32.6 weeks. The median overall survival was 30.5 months, with a two-year survival rate of 56%. This study demonstrates that local delivery of irinotecan into the brain parenchyma offers a safe route of administration over systemic delivery in the treatment of recurrent glioblastoma.

## 1. Introduction

Glioblastoma (GBM) is the most aggressive and invasive tumour of the central nervous system [1,2] and the most common malignant primary brain tumour in adults [3]. Due to the infiltrative and invasive nature of GBM, it is impossible to fully remove surgically, which means that recurrence almost always presents at the borders of the resection cavity despite high-dose radiation therapy. Additionally, there is no approved treatment option for recurrent patients. GBM represents 45.2% of primary malignant brain and central nervous system tumours [4], with a global annual incidence rate of 3.19 per 100,000 [5]. Despite various clinical trials exploring new treatments, the median survival for GBM patients is only 12 to 15 months, with a mere 5% surviving beyond five years [6]. An increasing number of patients with recurrent GBM are opting for re-operation to manage their disease when standard second-line therapies are ineffective, provided their performance status is still adequate [7,8].

Delivering chemotherapeutic drugs to the brain is challenging because of the blood–brain barrier (BBB) [9], which only allows the passage of low molecular weight, electrically neutral, and low hydrophobic drugs [10]. Consequently, most treatments for GBM are unsuccessful since chemotherapeutic agents are typically large, ionically charged, and hydrophilic, preventing them from crossing the BBB. Their administration via the intravenous or oral route cannot achieve the concentration levels required in the brain for therapeutic effect, resulting in the need for larger systemic doses [10,11], often with dose-limiting side-effects.

Local administration directly into the margin tissue of a resected GBM would deliver the chemotherapeutic drug directly to the residual tumour tissue, offering a number of advantages, like lower drug doses and fewer side effects, as it bypasses the systemic circulation [12]. This approach is logical since about 80 to 90% of GBM recurrences occur within 2 cm of the resection site [11]. Consequently, various local delivery approaches, including polymer millirods [13,14,15], seed [16,17], gels [18,19,20,21], and micro- and nanoparticle formulations [22,23,24], have been explored for direct administration into the margin of the resection cavity.

All clinical experience so far with local administration to the brain has been with the Gliadel^®^ wafers, a disc-shaped, 200 mg bioresorbable wafer containing 3.85% *w*/*w* of the chemotherapeutic drug carmustine, which received FDA approval in 1996 for the treatment of recurrent GBM [25,26]. Following the surgical removal of a GBM tumour, up to eight wafers are placed in apposition to the resected surface in the resection cavity, and the carmustine is released over a 5-day period [25]. The Gliadel wafers enable the delivery of a chemotherapeutic agent directly into the resection cavity and thus overcome the issues associated with BBB. Although initial clinical studies demonstrated a small but significant increase in survival [25,26,27,28] a Cochrane Review stated that when used in primary therapy, the Gliadel wafer extends survival without raising the incidence of adverse events. However, it does not seem to offer additional benefit for recurrent disease [29]. Additionally, only about one-third of GBM patients respond to carmustine treatment [30], and some patients experience cerebral edema as a significant adverse effect associated with Gliadel [31]. Widespread use of the Gliadel wafer has been limited due to the serious adverse events (surgical site infection, hydrocephalus, cysts in the resection cavity, acute hematoma, wound healing complications, and brain necrosis) associated with its use [32]. These adverse events are due to a number of reasons: (1) the wrong choice of drug for local delivery, and (2) the poor design of the device itself. Carmustine is a very toxic drug to local tissues, which makes it unsuitable for local delivery, particularly to the brain. The awkward shape of the wafers and the fact that they are placed into the cavity and not implanted into the brain tissue mean that tumour dosing relies on carmustine diffusion from the wafer into the brain tissue, restricting penetration distances to a few millimetres and thus the drug does not reach the deep-seated GBM cells. Furthermore, the carmustine released in the resection cavity is in direct contact with a weakened post-irradiated dura and the surgical wound, resulting in impaired healing and infection at the surgical site. In this study, we investigate the intraparenchymal administration of irinotecan hydrochloride (IRN) directly into the resection margin of recurrent GBM patients.

IRN is a semi-synthetic pro-drug [33], whose active metabolite 7-ethyl-10-hydroxycamptothecin (SN-38) inhibits the Topoisomerase I enzyme group [34]. These enzymes facilitate DNA transcription and replication by creating temporary breaks in one or both DNA strands, allowing them to uncoil [35]. Topoisomerase I binds covalently to DNA, forming a cleavable complex [35]. Sn-38 binds to this complex, preventing the enzyme from rejoining the DNA strands, resulting in S-phase-specific cell death [34,35,36]. IRN is used to treat advanced colorectal cancer in combination with 5-fluorouracil (5-FU) and folinic acid [37]. It has shown efficacy against GBM both as a monotherapy [38,39,40,41,42,43,44,45,46] and in combination with drugs such as Temozolomide (TMZ) [47,48], carmustine [49,50,51], and bevacizumab [52,53,54,55,56,57,58,59,60]. As a monotherapy, IRN demonstrated a response rate of 13 to 100% and progression-free survival of 3 to 12 months [36]. Despite its ability to cross the BBB, achieving therapeutic levels in the brain requires high intravenous doses of 125 to 500 mg/m^2^, leading to significant systemic side effects, including severe gastrointestinal toxicity, early and late-onset diarrhea, and severe neutropenia, which limits its use in GBM treatment [61].

We have previously demonstrated in preclinical models of GBM that the local administration of IRN directly into the tumour resection margin could improve therapeutic outcomes by allowing for the delivery of larger doses directly while reducing systemic concentrations and thus alleviating the side-effects [16,17]. Due to SN-38 being responsible for the side effects associated with IRN and the lack of a regulatory-approved SN-38 formulation suitable for local administration into the brain parenchyma, we decided to use the CE-approved DC Bead^®^ loaded with IRN (DEBIRI) from Boston Scientific. DEBIRI’s and DC Beads containing doxorubicin (DEBDOX) implanted into the brains of healthy and tumour bearing BD IX rats significantly improved survival compared to a placebo [62]. Although there was no significant difference in survival between DEBDOX and DEBIRI groups, DEBDOX was associated with local toxicity, causing notable hemorrhaging around the implantation site, whereas DEBIRI did not exhibit toxicity [62].

In this study, we report the intraparenchymal administration of IRN directly into the resection margin of recurrent GBM patients using the DEBIRI. The cytotoxicity of IRN was compared to SN-38 using six recurrent GBM tissue samples. Finally, IRN was loaded into the DEBs and administered directly into the brain parenchyma of the resection cavity, and the patients were monitored for steroid use, swelling at the resection site, IRN and SN-38 plasma concentrations, as well as survival.

## 2. Materials and Methods

### 2.1. Materials

IRN for in vitro work was purchased from LGM Pharma (Nashville, TN, USA). Dulbecco’s modified eagle medium (DMEM-F12), deoxyribonuclease I (DNA’s), sodium pyruvate, sodium bicarbonate solution (NaHCO_3_), fetal bovine serum (FBS), thiazolyl blue tetrazolium bromide (MTT) powder, dimethyl sulfoxide (DMSO), trypsin replacement enzyme 1X, were all purchased from Sigma Aldrich (Dorset, UK). Collagenase, trypan blue solution 0.4%, minimum essential medium (MEM), antibiotic-antimycotic (containing penicillin, streptomycin and fungizone), and hank’s balanced salt solution (HBSS) were all purchased from Gibco (Loughborough, UK). 1.077 +/− 0.001 g/mL Ficoll-Paque density gradient cushions were purchased from GE Healthcare Life Sciences (Chalfont St Giles, UK), Pronase from Roche Diagnostics (Indianapolis, IN, US) and phosphate-buffer saline (PBS) from Oxoid (Basingstoke, UK). GBM tissue samples were retrieved from patients who received resection surgery at the Queen Elizabeth Hospital, Birmingham, UK. GMP grade drug-eluting beads (DC Bead^®^) containing 100 mg of IRN (DEBIRI) were generously donated by Biocompatibles UK Limited (Farnham, UK).

### 2.2. Recurrent GBM Brain Tumour Tissue Collection and Cell Extraction

Tumour core tissue from recurrent GBM patients undergoing craniotomies at the Queen Elizabeth Hospital was collected under ethical approval (application number: 11-029) from the Human Biomaterials Resource Centre (HBRC). Upon collection, samples were immediately placed in collection fluid and transported to the lab. The tumour tissue was placed in HBSS (Invitrogen-Life Technologies, Carlsbad, CA, US), cut into 1 mm^3^ fragments, and washed with HBSS to remove excess blood clots. These fragments were then suspended in 30 mL of HBSS and subjected to enzymatic digestion using Collagenase (0.25 mg/mL; Invitrogen-Life Technologies, Carlsbad, CA, USA), Pronase (0.5 mg/mL; Roche, Basel, Switzerland), and DNase (0.4 mg/mL; Sigma-Aldrich, Dorset, UK) for 30 min at both 37 °C and 4 °C with constant stirring. Any remaining undigested material was filtered through 100 µm pore nylon mesh. The resulting suspension was layered onto two 12 mL Ficoll-Paque density gradient cushions (density: 1.077 ± 0.001 g/mL) and centrifuged at 400 g for 30 min at room temperature. Tumour cells, which formed a band at the interphase, were carefully extracted, while blood cells, forming a pellet, were discarded. The tumour cells were then resuspended in 15 mL of HBSS and centrifuged for 5 min at 1200× *g*. After removing the supernatant, the pellet was resuspended in 1 mL of HBSS for a viability check. Cell viability, assessed using the Trypan Blue exclusion method, ranged between 98 and 100%.

### 2.3. Primary GBM Cell Culture

Cells were seeded at a density of 2 × 10^5^ cell/cm^2^ in a culture medium composed of a 1:1 ratio of Dulbecco’s Modified Eagle’s Medium (DMEM) and Ham’s F-12 with L-glutamne and sodium bicarbonate (Sigma-Aldrich, Dorset, UK). This medium was supplemented with 10% Fetal Bovine Serum, 100 µM sodium pyruvate, 0.05 mM non-essential amino acids, and a 1% antibiotic-antimycotic solution containing penicillin, streptomycin and fungizone (Invitrogen-Life Technologies, Carlsbad, CA, US). The cells were incubated at 37 °C with 5% CO_2_, with the medium being refreshed every two days. Upon reaching confluence, the cells were passaged by removing the culture medium and adding trypsin (Sigma-Aldrich, Dorset, UK) (2.5 mL for 75 cm^3^ flasks and 1.5 mL for 25 cm^3^ flasks) to detach the cells. After detachment, 10 mL of fresh culture medium was added to the flask, and the cell suspension was transferred to a centrifuge tube. The suspension was centrifuged at 1000 rpm for 3 min, the supernatant was discarded, and the cell pellet was resuspended in culture medium and returned to the incubator at 37 °C with 5% CO_2_. The cells were further passaged twice to achieve the required cell numbers for cytotoxicity studies.

### 2.4. Cytotoxicity of IRN and SN-38 against Patient-Derived GBM Cells

Cells were seeded at 4000 cells/well into 96-well flat-bottomed microtiter plates and cultured with 200 µL of cell culture medium containing different concentrations (0, 0.0001, 0.001, 0.01, 0.1 and 1 µM) of IRN and SN-38 for five days. Cytotoxicity was assessed using the standard 3-(4,5-Dimethylthiazol-2-yl)-2,5-Diphenyltetrazolium Bromide (MTT) assay. All experiments were conducted in triplicate.

### 2.5. Clinical Trial: Intraparenchymal Administration of DEBIRI in Recurrent GBM Patients

In a first-in-man, trial a Phase 1 study of intraparenchymal therapy with DEBIRI as an adjunct therapy to the best standard of care in patients with recurrent surgically resectable GBM (NCT02433392) was performed. The aim of the study was to evaluate the safety of local injection of IRN into the wall of the resection cavity after tumour removal. In particular, we looked at using clinical assessment and imaging for any evidence that high-dose local delivery of IRN was toxic. Markers included clinical status, the use of dexamethasone, new or worsening seizures, and patient acceptability. A total of 9 out of 10 patients approached with recurrent GBM and a Karnovsky performance scale of 70% or better consented (Table 1). All had received previous surgery, radiation, and completed TMZ chemotherapy according to the Stupp regimen. 4 patients had undergone a second surgery, 7 had received conventional Procarbazine, CCNU, Vincristine (PCV) second-line chemotherapy and 7 had received levetiracetam or were currently taking this. There were 8 males 1 female with an average age of 54 years. MRI imaging confirmed unifocal disease recurrence (RANO criteria) considered suitable for resection at a Multidisciplinary Team (MDT) meeting. All patients underwent serial routine sets of liver function, electrolyte, and hematology counts. IDH1,1p19q, and MGMT data were available for these patients.

At operation, each patient’s tumour was maximally resected under the microscope to leave an apparent tumour-free, white matter-lined cavity. 3 mL vials of IRN(100 mg)-eluting DC beads^®^ (DEBIRI) suspended in viscous alginate (Biocompatibles, Farnham, UK) were drawn up into a standard syringe. Multiple (~30) 0.1 mL injections at a rate of 0.05–0.1 mL/30 s were carried out with a soft 24 G plastic catheter into the resection cavity wall to a depth of 0.3–0.5 cm under microscope vision. The viscous alginate suspension retained the beads until each track could be sealed with Evicel^®^ to retain the injectate. There were no perioperative complications with this simple technique, with dural and skin closure as normal. Patients were monitored closely as described and underwent MRI within 48 h. One patient developed mild hemiparesis post-surgery that resolved in 48 h. One patient on levetiracetam had a post-operative seizure. 1 patient remained in hospital for wound observation, not requiring intervention, and 1 patient had a delayed chest infection, requiring delayed discharge. The remaining patients were discharged after their MRI scans. All patients were reviewed at two weeks and then at two monthly intervals with imaging for 12 months. There were no hematological or metabolic issues, and all patients were positive about the process.

### 2.6. Statistical Analysis

Statistical analysis of the cell culture data was carried out using a one-way analysis of variance (ANOVA) with GraphPad Prism version 5.02 For Windows (GraphPad Software, San Diego, CA, USA). Post hoc mean comparisons were conducted using Tukey’s Honestly Significant Difference test. The statistical analysis in the clinical trial was purely descriptive to give insight into the feasibility and safety of DEBIRI administration into the brain parenchyma. No formal sample size calculation was performed, and the study was not statistically powered.

## 3. Results

### 3.1. Comparison of the Cytotoxicity of IRN and SN-38 Using Primary Cells from Recurrent GBM Patients

**Figure 1:**
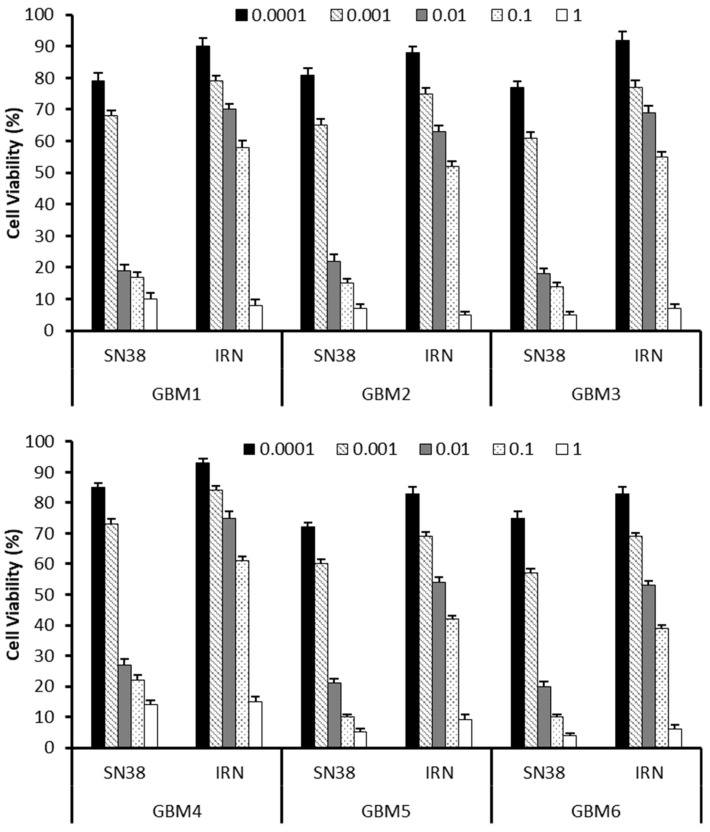
Comparison of the cytotoxicity of SN38 and IRN in six primary GBM cells at passage number 3 taken from recurrent GBM patients at a concentration range of 0.0001 to 1 µM.

IRN is a semi-synthetic pro-drug that is converted to its active metabolite SN-38 via the enzyme carboxylesterase [33]. We have previously demonstrated in preclinical models of GBM that the local administration of IRN directly into the tumour margin could enhance therapeutic outcomes by enabling the administration of higher doses directly to the site, thereby reducing systemic concentrations and minimizing side effects [16,17]. Wang et al. showed that IRN is metabolized into SN-38 following intratumoral delivery to glioma [63]. Figure 1 compares the cytotoxicity of SN-38 and IRN on the six primary cell samples at concentrations ranging from 0.0001 to 1 µM. For all cell samples, both SN-38 and IRN were shown to be cytotoxic across all concentrations, with cytotoxicity increasing with an increase in SN-38 and IRN concentrations (Figure 1). However, IRN’s cytotoxicity was significantly lower than that of SN-38 at concentrations below 1 µM (*p* values = 0.040, 0.035, 0.005, 0.004, respectively). IRN is known to be cytotoxic without conversion to SN-38 [64] and was shown to reduce cell viability by 7 to 17%, 9 to 31%, 25 to 45% and 39 to 61% across the six cell samples at a concentration of 0.0001, 0.001, 0.01 and 0.1 µM, respectively (Figure 1). Whereas SN-38 reduced the cell viability by 15 to 28%, 27 to 43%, 73 to 82% and 78 to 90% across the six cell samples at a concentration of 0.0001, 0.001, 0.01 and 0.1 µM, respectively (Figure 1). At a concentration of 1 µM the IRN has a similar cyto (*p* = 0.12) toxicity to that of SN-38 with both reducing cell viability by 85 to 95% and 86 and 96%, respectively, across the six cell samples.

### 3.2. Steroid Use and Swelling in Recurrent GBM Patients after Intraparenchymal Administration of IRN Directly into the Resection Margin Compared to Patients Administered the Gliadel Wafer

In GBM patients, steroids are used for the treatment of brain edema and associated symptoms. High-dose glucocorticoids reduce cerebral edema and can improve headaches and other neurologic deficits caused by vasogenic edema. In patients who present with moderate to severe symptoms or who have a risk of herniation, an initial dose of 8 mg once or twice per day of dexamethasone is normally used. For asymptomatic patients, steroids are not required; however, a minimal dose is normally used, especially if other treatments such as radiotherapy may make the edema worse. Increased steroid use has been noted with the use of the Gliadel wafers [31,65]. Glucocorticoids are associated with several side effects, such as serious infections, sepsis, and thrombosis, while Pitter et al. have suggested that the use of corticosteroids can reduce survival in GBM patients [66].

**Figure 2:**
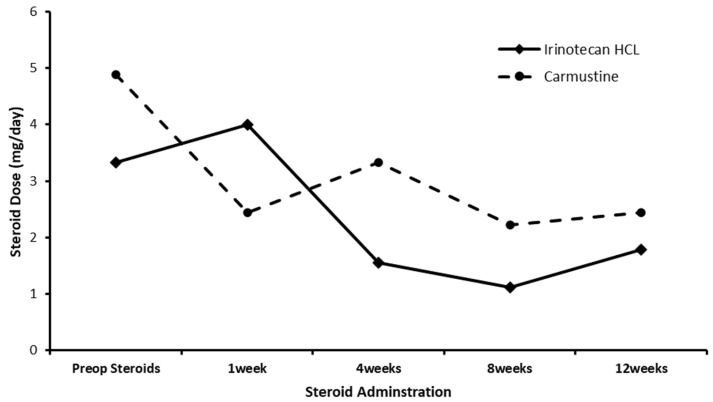
Steroid use in recurrent GBM pre- and post-treatment with either the carmustine wafer or the irinotecan (100 mg)-eluting beads (DEBIRI).

Figure 2 compares the average dose of dexamethasone for those patients receiving intraparenchymal administration of IRN directly into the resection margin to a historical control of patients administered the Gliadel wafer [66]. Higher pre-op steroid doses in the carmustine group compared to the IRN group resulted in lower average steroid doses in the carmustine group 1-week post-op. However, by 4 weeks post-op, the average steroid dose in the IRN group was much lower than the carmustine group. The average daily dose of dexamethasone from 4 weeks post-op to 12 weeks post-op in the IRN group was 1.4 mg per day compared to 2.67 mg per day for the carmustine group. We appreciate that the inter-trial comparison of steroid use in this trial and previously reported Gliadel wafer trials has limitations due to the difference in numbers of patients and the different eligibility criteria. However, convulsions, cerebral edema, and intracranial hypertension are known complications of Gliadel use, which are monitored and controlled with the use of steroids.

**Figure 3:**
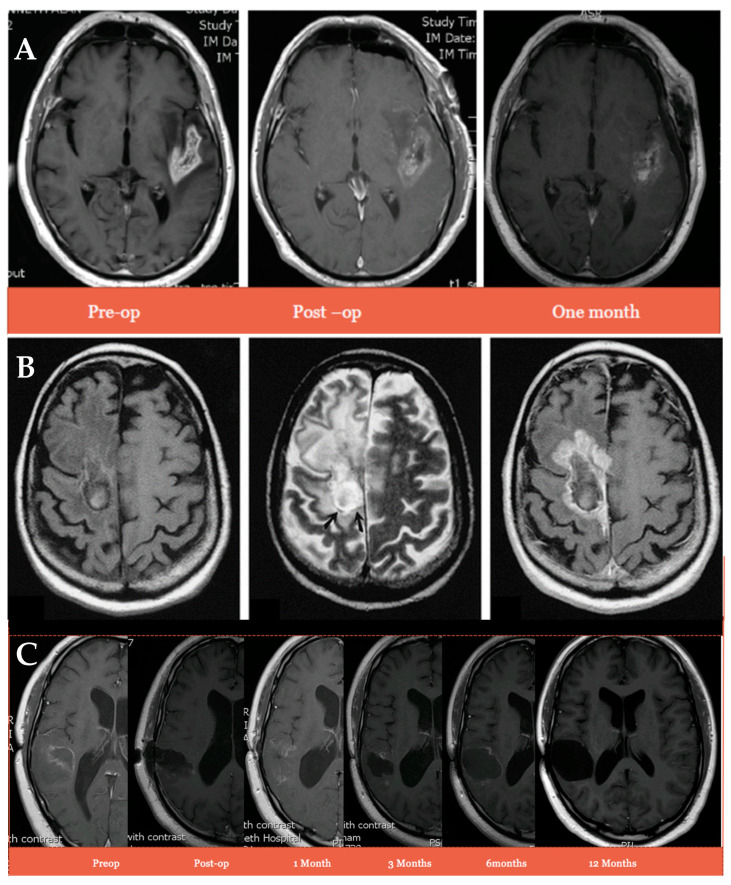
Comparison of MRI scans from a patient treated locally with irinotecan (100 mg)-eluting beads (**A**) and carmustine wafer (**B**) pre-op, post-op, and one month after local administration. A 29 year old male with a right temporal GBM resected and cavity wall injected with irinotecan (100 mg)-eluting beads (**C**). Scans show pre-op, post-op, 1 month, 3 months, 6 months, and 12 months post-injection.

Despite the lower dose of dexamethasone in the IRN study, there was an absence of swelling, inflammation, or any suggestion of pseudo-abscess formation (Figure 3A) compared to that normally seen with the Gliadel wafers (Figure 3B) [67]. Furthermore, patient 2, who was still alive 667 days post-surgery, had no signs of swelling 12 months post-administration of the IRN (Figure 3C). This indicates a much less toxic local reaction from tissue and residual tumour, resulting in the lower need for steroids and reduced risk of infection, wound breakdown, and the need for decompressive reoperation.

### 3.3. IRN and SN-38 Plasma Concentration after Intraparenchymal Administration of IRN Directly into the Resection Margin

**Figure 4:**
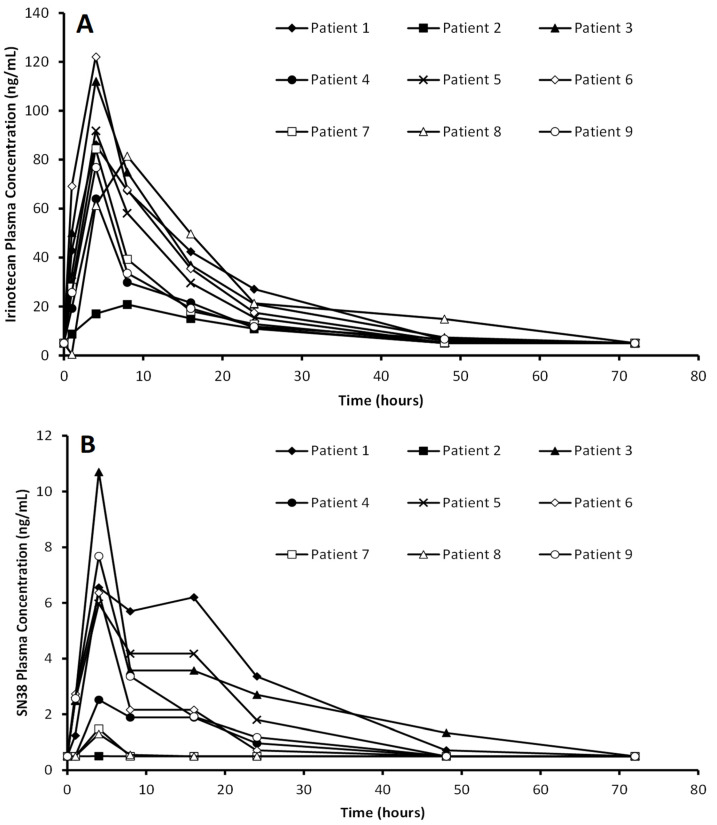
Irinotecan (**A**) and SN38 (**B**) plasma concentrations from patients treated locally with irinotecan (100 mg)-eluting beads.

The plasma concentration of both IRN and SN-38 after intraparenchymal administration of the DEBIRI is presented in Figure 4A and Figure 4B, respectively. The IRN is off-loaded much quicker than expected, with maximum plasma concentrations detected between 4 and 8 h after administration, which is due to the fast release of the IRN from the DEBIRI [62]. Despite the quick clearance of IRN from the brain, the plasma levels of IRN after local administration are between 8.2 and 48 times lower when compared to IV administration of a 125 mg/m^2^ dose, which typically has plasma levels averaging 1000 ng/mL [68]. The detection of SN-38 in the plasma confirms the in vitro data (Figure 1) that IRN is converted to SN-38 by GBM tissue. The SN-38 plasma concentrations were between 11.2 and 92.3 times lower when compared to an average of 120 ng/mL normally detected after IV administration [68]. These significantly lower IRN and SN-38 plasma levels explain why none of the patients suffered from any of the typical side effects associated with systemic administration of IRN, such as gastrointestinal toxicity, diarrhea, and severe neutropenia.

### 3.4. The Impact of Intraparenchymal Administration of IRN Directly into the Resection Margin on the Survival of Recurrent GBM Patients

**Figure 5:**
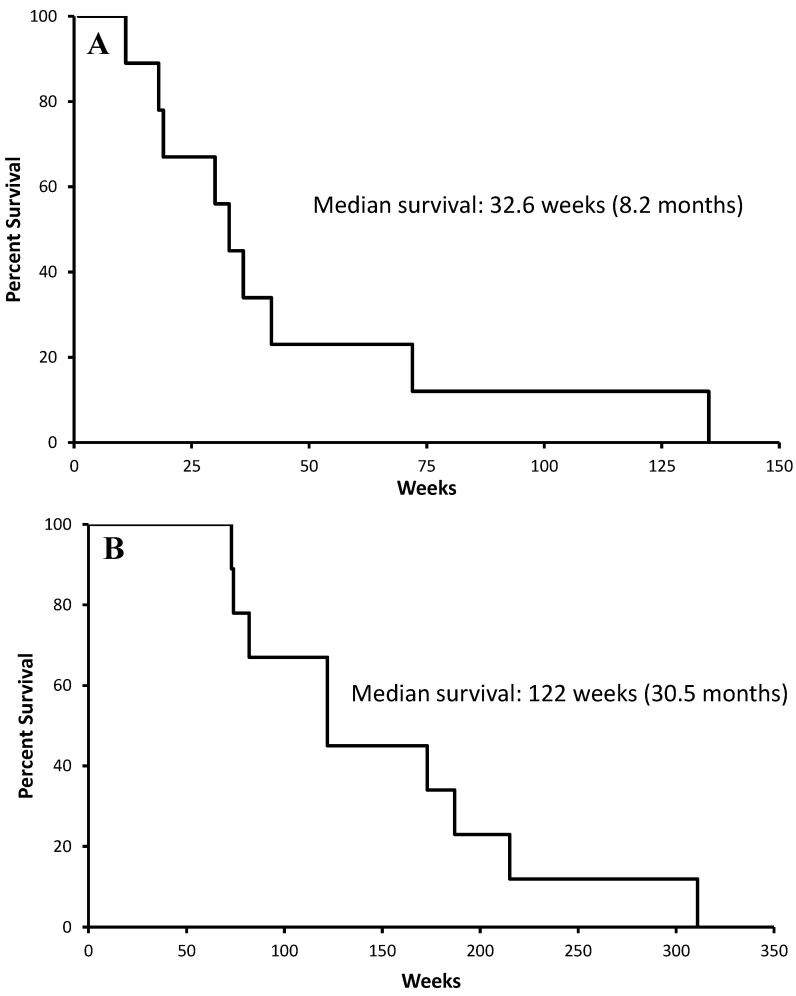
Patient survival post irinotecan (100 mg)-eluting bead administration (**A**) and overall post initial diagnosis (**B**).

Figure 5A,B show the survival from intraparenchymal administration of the DEBIRI and overall survival from GBM diagnosis, respectively. In this small multiply-treated patient group, the median survival from DEBIRI administration was 32.6 weeks, which is similar to the 31 weeks for the Gliadel wafers [27], while the medial overall survival from the initial diagnosis was 122 weeks (30.5 months). However, three patients in this group had IDH-mutated tumours, which are known to have a better prognosis than IDH wildtype tumours. When their survival data are removed from the analysis the median survival from DEBIRI administration and median overall survival from initial diagnosis reduce to 24.2 weeks and 89.6 weeks (21.3 months), respectively.

## 4. Discussion

IRN and SN-38 were cytotoxic to all six patient samples across all concentrations; however, IRN’s cytotoxicity was lower than that of SN-38 below 1 µM. This is due to enzyme kinetics, whereby an increase in the substrate (IRN) results in an increase in the product (SN-38). When the concentration of IRN is increased to 1 µM, its conversion to SN-38 is increased, which reduces the difference in cytotoxicity between IRN and SN-38. This supports the investigation of the intraparenchymal administration of IRN direct into the resection cavity of recurrent GBM patients.

We have demonstrated that the intraparenchymal administration of IRN directly into the resection margin of heavily treated recurrent GBM patients was safe. There was good wound healing with an absence of swelling, inflammation, or any suggestion of pseudo-abscess formation compared to the pattern normally seen with Gliadel wafers, indicating much reduced local toxicity and reduced risk of infection. This was reflected in the much lower requirement for concurrent use of debilitating dexamethasone when compared to Gliadel implanted patients. This is because IRN is less toxic than carmustine, making it more appropriate for local delivery to the brain, and it predominately is being administered directly into the brain parenchyma rather than the cavity. Additionally, none of the patients exhibited the typical systemic toxicities associated with IRN, which is due to the peak plasma levels of IRN and SN-38 being ten-fold lower than those reported for IV infusions and well below the systemic toxicity threshold. The median overall survival in this select group of multiply treated patients was 32.6 weeks post DEBIRI administration and 122 weeks (30.5 months) post diagnosis. However, when the three patients with IDH-mutated tumours are removed from the survival data, the median survival decreased to 24.2 weeks and 89.6 weeks (21.3 months), respectively. Although our study demonstrated the safety and feasibility of intraparenchymal administration of DEBIRI, a significant limitation was the study’s lack of statistical power and the absence of a comparison group, preventing a definitive assessment of survival benefits.

## 5. Conclusions

This study indicates that administering IRN directly into the brain parenchyma provided a safer route of administration compared to systemic delivery for treating recurrent GBM. The offloading curves indicate an offload half-life of 10–20 h with the high local dose well tolerated. Based on these findings, the use of an enhanced implantable depot drug delivery formulation capable of proving sustained release of IRN for at least 1 week would allow for IRN levels to be maintained at therapeutic concentrations for longer, improving the clinical performance further. Given the lack of both local and systemic toxicity observed in this study, we believe that such an approach could be used in the primary setting, as a concurrent treatment to the current standard of care. Such an approach would ensure that patients are being treated in the window between surgery and radiation/systemic chemotherapy starting.

## Figures and Tables

**Table 1:** Patient age, sex, Karnofsky score, number of prior surgeries, prior chemotherapy regimen, IDH status, anti-epileptic drug and use of dexamethasone.

Patient	Age	Sex	Karnofsky Score	Prior Surgery	Prior Chemotherapy	IDH	MGMT Status	AED	Dexamethasone
1	51	M	100	×2	2× TMZ, 1× PCV	Mutant	Unmethylated	Lev	No
2	29	M	100	×1	1× TMZ, 1× PCV	Mutant	Methylated	Lev	Yes
3	56	M	90	×2	1× TMZ	Wild type	Methylated	Lev	No
4	73	M	80	×1	1× PCV	Wild type	Unmethylated	Lev	Yes
5	60	F	100	×1	1× PCV	Wild type	Methylated	Lev	Yes
6	63	M	80	×1	1× TMZ, 1× PCV	Wild type	Unmethylated	none	Yes
7	44	M	90	×2	1× TMZ	Mutant	Methylated	none	No
8	53	M	80	×2	1× PCV	Wild type	Methylated	Lev	Yes
9	63	M	90	×1	1× TMZ, 1× PCV	Wild type	Unmethylated	Lev	Yes

## Data Availability

The datasets generated during and/or analyzed during the current study are available from the corresponding author on reasonable request.
